# Polychromatic metasurfaces for complete control of phase and polarization in the mid-infrared

**DOI:** 10.1038/s41377-023-01257-5

**Published:** 2023-10-08

**Authors:** You Zhou, Jonathan A. Fan

**Affiliations:** Department of Electrical Engineering, Stanford University, Stanford, CA 94305 USA

**Keywords:** Optical materials and structures, Applied optics

## Abstract

Multifunctional metasurfaces based on wavelength-decoupled supercells are experimentally demonstrated, enabling new regimes of optical control for arbitrary orthogonal polarizations at different wavelengths.

Polarization and wavelength are fundamental properties of light that carry valuable details about the material composition, morphology, and texture of objects within a scene. Optical imaging systems that can detect these properties have broad applications in environmental monitoring, medical diagnostics, remote sensing, and space-to-ground communications. Conventional techniques for spectropolarimetry typically require a series of standard optical elements, and these systems are typically bulky with restricted design flexibility. To overcome these limitations in size, weight, and capability, metasurface optics^1^ capable of producing customized phase, amplitude, and polarization responses offer great promise. To date, a wide range of devices have been realized where combinations of wavelength^2^, angle^3^, spatial mode^4^, and polarization^5^ functionality can be simultaneously controlled. However, completely independent and arbitrary phase and polarization control for three or more wavelengths has remained elusive.

In a new publication, Chen and coworkers report the development of polychromatic metasurfaces for more comprehensive control of polarization and phase in the mid-infrared^6^. The ultracompact metadevices, fabricated on an all-silicon wafer, mimic the functions of filters, polarization beam splitters, and phase plates. The metasurfaces feature multifunctional supercells each consisting of four elliptically-shaped meta-atoms with adjustable dimensions and orientations (Fig. 1). While the utilization of meta-atoms to control polarization and chromatic response is well established, the reported combination of high aspect ratio meta-atom manufacturing together with the inverse design of meta-atom supercells enables access to new regimes of polychromatic operation. In particular, the authors expand the design space of conventional high aspect ratio nanopillar and via-hole-based metasurfaces^7,8^ using evolutionary optimization algorithms that produce supercell parameters featuring maximal transmission and isolation between different polarization and wavelength channels. The authors also develop a low-temperature deep etching technique to precisely define the silicon nanostructures within the stringent fabrication tolerances required by the high-aspect-ratio meta-atom configurations.

**Fig. 1:**
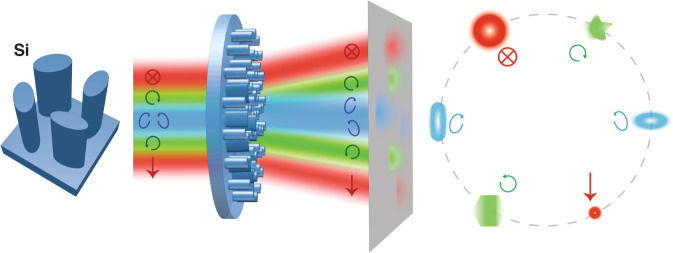
Schematic of the polychromatic metasurface. The metasurface employs wavelength-decoupled supercells consisting of four high aspect ratio elliptical meta-atoms. The size and orientation of each meta-atom can be independently adjusted, which enables independent control of phase for arbitrary orthogonal polarization bases at different wavelengths

Exploiting this platform, the authors showcase a series of multifunctional metadevices that allow complete control of phase and polarization at predefined multiple wavelengths. They demonstrate a multi-channel metalens that focuses light of three distinct wavelengths into six focal points, with pairs of points corresponding to linear, elliptical, and circular polarization bases. The platform is further extended to more complex wavefront control in which the authors design a metadevice that performs focusing and vortex beam generation, where the point spread functions can carry distinct topological charges for each polarization and wavelength channel. The authors additionally design a metadevice serving as an imaging filter that transmits at specific wavelengths and polarization states.

Metasurfaces supporting full-polarization control at multiple wavelengths can lead to new application opportunities for metasurfaces in the domains of sensing, encryption and polarimetric imaging. For example, by breaking the eigen-polarization constraint, multiple orthogonal polarization bases can be integrated on a single device without spatial interleaving^9^, leading to new classes of snapshot, hyperspectral polarization cameras^10^. Additionally, the platform can be adapted for gas sensing applications. By integrating gas-sensitive media with a polychromatic holographic metasurface, it becomes possible to generate distinct visual readouts for gas exposure at multiple wavelengths, each aligned with the absorption band of the specific gas of interest^11^. Moreover, meta-atoms with high aspect ratios are intrinsically desirable in the design of multifunctional devices: they can broaden the scope of wavefront engineering responses through the specification of large chromatic group delays^7,12^ or support complete control over amplitude, phase, and polarization in a wavelength-decoupled manner^13^. Pathways to dynamically controlling these optical properties are accessible by incorporating phase change materials, liquid crystals or other nonlinear materials to this metasurface platform^14^.

Looking ahead, the hybridization of the reported concepts with other complementary design and fabrication concepts suggests pathways to even further enhance multifunctional metasurface multiplexing capabilities. In one direction, nonlocality can be employed to further broaden the capabilities of dispersion engineering, using for example perturbation-based symmetry breaking^15^ to achieve independent wavefront control at discrete narrowband wavelengths. In another direction, limitations posed by the use of a single layer of meta-atoms can be potentially addressed using multi-layered metasurfaces^16^. We also anticipate that high resolution three-dimensional printing methods, such as two-photon polymerization lithography^17^, can be employed to enable volumetric mid-infrared metamaterials at submicron resolution. To mitigate reflection losses from the use of high-index substrates, membrane-based fabrication techniques can lead to free-standing nanostructures that offer high optical transparency^8,18^. From the perspective of inverse design, the reported design method relies on simply shaped meta-atoms guided by physical intuition, resulting in a limited design space. More advanced freeform topology optimization algorithms^19^ can unlock a much broader design space, leading to greater functional density and superior performance within a more compact footprint.
